# RETRACTED: Frolli et al. Executive Functions and Foreign Language Learning. *Pediatr. Rep.* 2022, *14*, 450–456

**DOI:** 10.3390/pediatric18040105

**Published:** 2026-08-05

**Authors:** Alessandro Frolli, Francesco Cerciello, Clara Esposito, Sonia Ciotola, Gaia De Candia, Maria Carla Ricci, Maria Grazia Russo

**Affiliations:** 1 Disability Research Centre, University of International Studies in Rome, Via Cristoforo Colombo 200, 00147 Rome, Italy; g.decandia1@studenti.unint.eu (G.D.C.); m.ricci@unint.eu (M.C.R.); mariagrazia.russo@unint.eu (M.G.R.); 2 FINDS-Italian Neuroscience and Developmental Disorders Foundation, 81040 Caserta, Italy; francesco.cerciello@fondazionefinds.it (F.C.); clara.esposito@fondazionefinds.it (C.E.); sonia.ciotola@fondazionefinds.it (S.C.)

The journal retracts the article “*Executive functions and foreign language learning*” [1] cited above.

Following publication, concerns were raised with the Editorial Office regarding the statistical analysis and ethical approval information reported in this article [1].

In accordance with the journal’s standard procedures, the Editorial Office and Editorial Board conducted an investigation during which questions were raised about the lack of appropriate Institutional Review Board approval prior to participant enrolment.

The authors cooperated during the investigation and proposed a Correction, providing clarifications relating to the statistical analysis and additional information relating to the ethical approval. However, after careful consideration, the Editorial Board determined that the explanations and supporting documentation did not sufficiently address the remaining concerns about the ethical approval information and concluded that the available documentation did not demonstrate compliance with MDPI’s policies for research involving human subjects, particularly regarding appropriate ethics approval prior to participant enrolment. As a result, the Editorial Board has decided to retract this paper as per MDPI’s retraction policy.

This retraction was approved by the Editor-in-Chief of the journal *Pediatric Reports*.

The authors have been informed of this retraction and have indicated their disagreement with this decision.
